# New variants of known folds: do they bring new biology?

**DOI:** 10.1107/S1744309110013242

**Published:** 2010-07-06

**Authors:** Eugene V. Koonin

**Affiliations:** a National Center for Biotechnology Information, National Institutes of Health, Bethesda, Maryland 20894, USA

**Keywords:** new variants of known folds

## Abstract

New distinct versions of known protein folds provide a powerful means of protein-function prediction that complements sequence and genomic context analysis. These structures do not supplant direct biochemical experiments, but are indispensable for the complete characterization of proteins.

## Abstract 2

New distinct versions of known protein folds provide a powerful means of protein-function prediction that complements sequence and genomic context analysis.

## 1. Introduction

Structural biology in general and structural genomic initiatives in particular face distinct challenges and yield different kinds of information depending on the novelty level of the solved structures. In the early days of structural genomics and related protein-structure initiatives, there was a strong emphasis on solving the structures of proteins whose fold could not be predicted from the sequence in order to obtain a comprehensive sampling of protein-structure space (Brenner, 2001 ▶; Nair *et al.*, 2009 ▶). Beyond doubt this remains a lofty goal, but we now seem to have approached the long tail of the fold-abundance distribution, so new folds are rarely discovered and most of those that do appear in structural studies are quite rare in nature (Jaroszewski *et al.*, 2009 ▶). Thus, the conceptual importance of the new fold hunt notwithstanding, the biological impact of fold discoveries is relatively small and continues to diminish. At the opposite end of the spectrum are structures that are closely related to already known ones, sometimes mutants. The study of closely related structures can help to elucidate the fine details of catalytic and binding mechanisms, particularly when the structures of proteins complexed with sub­strates and ligands are solved. The middle ground belongs to structures that are significant variations of known folds. Realistically, this is the most common class of findings accessible to structural genomics and related large-scale projects, such as PSI, especially if the targets are preselected for diversity (Dessailly *et al.*, 2009 ▶). How informative and illuminating are these structures? Or, more precisely, how much unique information can one derive from an actual experimental structure above and beyond what can be gleaned from sequence analysis? These are far from being idle questions because the answers are crucial for the choice of optimal strategies in large-scale structure-determination projects. The four articles in this section describing variants of known folds provide ample material to address these issues (Table 1 ▶).

## 2. Structures that are variants of known folds and biological implications

The article by Xu *et al.* (2010 ▶) reports the structure of the ortholog of the essential bacterial protein YeaZ from the hyperthermophilic bacterium *Thermotoga maritima* (TM0874). In a testimony to the rapid pace and considerable parallelism of structural genomic efforts, this is already the third reported structure of a YeaZ ortholog; the first two structures are those from *Escherichia coli* (Jeudy *et al.*, 2005 ▶) and *Salmonella typhimurium* (Nichols *et al.*, 2006 ▶) YeaZ. The TM0874 sequence is 23–24% identical to those of the *E. coli* and *S. typhimurium* orthologs. Thus, technically, the *Thermotoga* structure adds only variation within a protein family (as defined in SCOP; Andreeva *et al.*, 2008 ▶). This apart, it is interesting to discuss the novelty brought about by the YeaZ structures taken together. This protein belongs to the ASKHA (acetate and sugar kinase/HSP70/actin) ATPase superfamily of the RNAse H fold (Aravind & Koonin, 1999 ▶; Hurley, 1996 ▶). More precisely, YeaZ is an ancient widespread paralog of another essential and ubiquitous protein, YgjD, which is the last protein in the ‘universal core of cellular life’ for which the function remains un­known; in a sense, it is the top target of functional genomics as far as individual genes are concerned (Galperin & Koonin, 2004 ▶). The search for the function(s) of YgjD and its orthologs (including the best characterized eukaryotic Kae1) has been a long and tangled quest. Originally, on the basis of the prediction of the ASKHA fold, the presence of an insert resembling the active site of Zn-dependent proteases and some experimental data suggestive of protease activity, it was proposed that YgjD is an ATP-dependent metalloprotease, possibly with chaperone activity (Aravind & Koonin, 1999 ▶). Genome-context analysis suggested a role of this protein in translation (Wolf *et al.*, 2001 ▶). Biochemical experiments confirmed the ATPase activity of YgjD, but not the protease activity, and also implicated YgjD in DNA repair (Hecker *et al.*, 2007 ▶). As for YeaZ, this protein lacks the metal-binding insert but retains the ATP-binding motifs, so it was characterized as an inactivated protease that potentially retains ATPase activity (Wolf *et al.*, 2001 ▶). Finally, both comparative genomic context analysis (Wolf *et al.*, 2001 ▶) and recent proteomic studies (Handford *et al.*, 2009 ▶) suggest that YgjD and YeaZ belong to the same network of proteins with linked functions. Thus, it has been hypothesized that YgjD and YeaZ are subunits of a still uncharacterized chaperone complex with a function related to translation (Wolf *et al.*, 2001 ▶). Against this rich background, what is the unique contribution of the YeaZ structure? Not at all unexpectedly, the structures confirm the ASKHA fold prediction (Jeudy *et al.*, 2005 ▶; Nichols *et al.*, 2006 ▶; Xu *et al.*, 2010 ▶). More importantly, however, careful examination of the structure suggests that the YeaZ-family proteins are very unlikely to bind ATP or any other nucleotide (Xu *et al.*, 2010 ▶). The ASKHA fold consists of a tandem duplication of RNAase H domains. In the YeaZ family, the distal RNAse H domain is truncated and the two domains are oriented in such a manner that nucleotide binding does not appear to be possible. A tempting hypothesis prompted by these findings is that YeaZ could be a regulator of the ATPase activity of YgjD (Xu *et al.*, 2010 ▶). In addition, the structural analysis of Xu and coworkers predicts the surface of the YeaZ molecule that is likely to mediate interactions with other proteins, possibly YgjD. Thus, the structure is beyond doubt a useful contribution to the elucidation of the still enigmatic, but probably central, bacterial cell functions of YeaZ, YgjD and their complexes. Admittedly, however, it is only an intermediate step: the solution remains to be reached in direct biochemical experiments.

The article by Han *et al.* (2010 ▶) reports the structure of another NTP hydrolase that presents a stark contrast to YeaZ both structurally and functionally. This protein, YP_001813558.1, comes from the rather exotic extremophilic bacterium *Exiguobacterium sibiricum* isolated from Siberian permafrost and is a member of the superfamily of all-α-helical NTP pyrophosphohydrolases that is distantly related to the other families in this superfamily, including MazG (another NTP pyrophosphohydrolase), dimeric dUTPases and phospho­ribosyl-ATP pyrophosphohydrolases (PRA-PH). The new structure shares with all these proteins a structural core which comprises a four-helical bundle and the general configuration of the active site, but otherwise shows unique features. Firstly, the *E. sibiricum* protein contains about twice as many amino-acid residues as the other enzymes in the same superfamily owing primarily to the presence of two long additional helices. Secondly, although many proteins in this superfamily form dimers or tetramers (Moroz *et al.*, 2005 ▶), the new structure shows an unusual segment swapping between the two monomers. Han and coworkers tentatively link this unique structural feature to the psychrophilic lifestyle of the bacterium from which the protein was isolated, an intriguing but so far speculative possibility. With regard to the function of YP_001813558.1, a close inspection of the predicted catalytic site suggests that, similar to MazG, this protein could be specific for dNTP. Both the MazG and dUTPase families of the all-α NTP pyrophosphohydrolase superfamily belong to the broad class of ‘house-cleaning’ enzymes whose function in the cell is to eliminate deleterious noncanonical NTPs such as dUTP (Galperin *et al.*, 2006 ▶). Certainly, it is tempting to hypothesize that the protein from *E. sibiricum* has the same type of function. This possibility seems particularly plausible considering that in the closest homologs of YP_001813558.1 (*e.g.* AAN59453.1 from *Streptococcus mutans*) the NTP-phosphohydrolase domain is fused to a hydrolase domain of the HAD superfamily, which also includes a variety of house-cleaning enzymes (Kuznetsova *et al.*, 2006 ▶).

Kumar *et al.* (2010 ▶) report the structures of two orthologous small proteins with unknown functions from different species of the bacterium *Shewanella*. These proteins represent a conundrum that has become quite common with the advance of massive genome sequencing, in particular of bacteria and archaea: comparative genomic analysis yields a large family of small proteins that are conserved in a broad variety of prokaryotes and adopt a globular conformation on the basis of prediction and/or structure determination, but have no known function or even strong functional clues. Often, detailed sequence and structural comparisons indicate that these small globular domains bind various small-molecule ligands and the resulting conformation change contributes to regulation of enzyme activity or signal transduction; these ligand-binding domains are found either as fusions with various other enzymatic, transport and regulatory domains or are solo (Anantharaman *et al.*, 2001 ▶). In all likelihood, this is the case with the two *Shewanella* proteins studied by Kumar and coworkers. The structures of these proteins reveal similarity to the structures of two distantly related superfamilies of ligand-binding domains, namely the SpoIIAA-like bacterial domains known to bind nucleotides (in particular flavin derivatives; Aravind & Koonin, 2000 ▶) and the CRAL-TRIO domains, which are carriers of diverse nonpolar substances including lipids (Panagabko *et al.*, 2003 ▶). The *Shewanella* proteins studied by Kumar and coworkers possess cavities that could accommodate various small molecules. Thus, considering the similarity to the CRAL-TRIO domains, in particular in the shape of the cavity, Kumar and coworkers hypothesize that this domain is a carrier of nonpolar molecules and is likely to function in a membrane-dependent manner given the presence of two long amphipathic α-helices that would peripherally bind to membranes (Kumar *et al.*, 2010 ▶). Exhaustive *PSI-BLAST* searches (Altschul *et al.*, 1997 ▶) detected homologs of this domain in numerous methyl-accepting chemotaxis proteins and other proteins that are involved in signal transduction from diverse bacteria (not mentioned by Kumar and coworkers; E. V. Koonin, unpublished work), suggesting that the new domain also contributes to signal transduction. Arguably, the most surprising finding of Kumar and coworkers is that the two proteins whose structures they report assume different conformations despite 54% sequence identity. The YP_001095227.1 protein from *S. loihica* is in the open conformation, with the two long α-helices exposed and the cavity available to accommodate the ligand; in contrast, the YP_749275.1 protein from *S. frigidimarina* adopts the closed con­formation, with the α-helices packed and obstructing access to the cavity. From the observation of the two distinct conformations of these proteins, Kumar and coworkers develop a plausible hypothesis on their mode of function: it is proposed that these proteins form water-soluble dimers in the closed conformation, but membrane interaction induces a switch to the open ligand-binding conformation (Kumar *et al.*, 2010 ▶). Thus, the conformation transition suggested by the comparison of the two solved structures of orthologous proteins is likely to be the basis of the function of these proteins.

The work of Das *et al.* (2010 ▶) presents the structure of the uncharacterized lipoprotein KPN03535 from the opportunistic pathogenic bacterium *Klebsiella pneumoniae*, illustrating a very different facet of structural genomics. There are no readily detectable homologs of this protein in organisms other than *Klebsiella*. How­ever, using an advanced fold-recognition approach, Ginalski and coworkers found that this protein belongs to a distinct family of bacterial oligomer-binding fold (OB-fold) domains (BOF) that are present in diverse secreted bacterial proteins (Ginalski *et al.*, 2004 ▶). Das and coworkers confirm this nontrivial prediction and take it a step further through a detailed analysis of the structural similarities between KPN03535 and other OB-fold domains (Das *et al.*, 2010 ▶). OB-fold domains are numerous and enormously diverse (Arcus, 2002 ▶) and show a wide spectrum of binding specificities, but Das and coworkers specifically predict that KPN03535 is a nucleic acid-binding protein on the basis of the substantial similarity of the solved structure to the structures of single-stranded DNA-binding proteins. The specific function of the protein, however, remains unknown.

## 3. Concluding remarks

**Figure 1:**
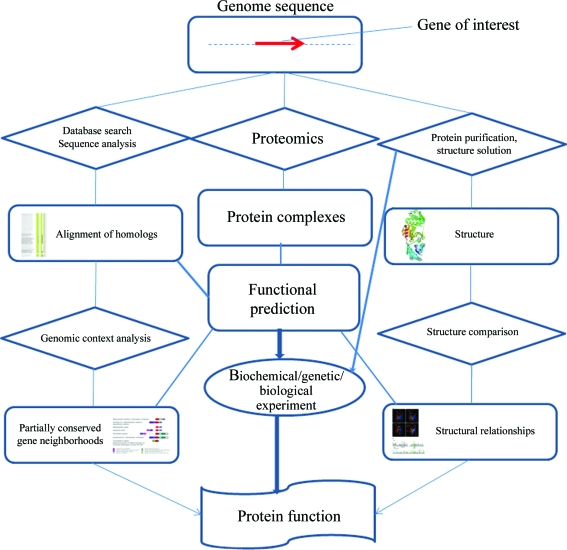
From genome sequence to protein function: the interconnected pipelines of protein sequence, structure and function analysis. The lines and arrows connecting modules schematically denote the flow of information and/or materials. The weight of the lines roughly reflects the relative contribution of the respective type of data to the functional characterization of a protein.

So what is the impact of these structures which are new variants of known folds? The structures do no magic: the functions of uncharacterized proteins are not instantaneously understood. Nevertheless, the utility of the increasing diversity of fold representation in the structure databases is clear and substantial. Essentially, these structures provide a means of functional prediction that extends and complements the predictions made by sequence comparison and genomic context analysis (Table 1 ▶). In some cases, when the sequences are highly conserved and the protein in question is common enough for context analysis to be highly informative, the added value of the structure is only incremental (the case of YeaZ). On other occasions, such as the discovery of an OB-fold in a *Klebsiella* lipoprotein, structural clues can be decisive, given that the protein sequence and context are poorly conserved. Furthermore, structural analysis has the potential to produce truly unique information such as the segmental swap in the dimer structure of the psychrophilic NTP-pyrophosphohydrolase or the two alternative conformations of the ligand-binding proteins from *Shewanella*. Therefore, to conclude with a generalization, the comprehensive characterization of a protein’s function proceeds through a network of computational and experimental pipelines: sequence–genomic context–structure–proteomics–biochemistry (Fig. 1 ▶); the pipeline is modular, so that the order of the modules can be switched and the connections between them rewired, but each is essential.

## Figures and Tables

**Table 1:** New structures that are variants of known folds and their biological impact

Protein/PDB code/organism	Fold, superfamily	Known or predicted function(s)	Impact of the new structure	References and comments
TM0874 (YeaZ)/2a6a/*Thermotoga maritima*	RNAse H fold, ASKHA superfamily	Part of a molecular chaperone (?) complex with the paralog YgjD, possible role in translation	Structure suggests that YeaZ does not bind ATP; putative regulator of YgjD; novel interaction surfaces predicted	Xu *et al.* (2010 ▶); two structures from mesophilic bacteria are also available (Jeudy *et al.*, 2005 ▶; Nichols *et al.*, 2006 ▶)
YP_001813558.1/2rfp/*Exiguobacterium sibiricum*	All-α-helical NTP pyrophosphohydrolase fold/superfamily	NTP pyrophosphohydrolase, putative house-cleaning enzyme	Unique structural features including domain swapping, possibly related to psychrophily	Han *et al.* (2010 ▶)
YP_001095227.1/2q3l/*Shewanella loihica*, YP_749275.1/2ook/*S. frigidimarina*	SpoIIAA-like fold/superfamily	Small-molecule binding, lipid binding, regulatory functions	Comparison of the two structures suggest a functionally important conformation switch	Kumar *et al.* (2010 ▶)
KPN03535/3f1z/*Klebsiella pneumoniae*	OB-fold, novel superfamily (BOF)	Secreted lipoprotein, probably nucleic acid-binding	Nucleic acid properties predicted solely from structure	Das *et al.* (2010 ▶)
